# Exploring the Terminal Pathway of Sex Pheromone Biosynthesis and Metabolism in the Silkworm

**DOI:** 10.3390/insects12121062

**Published:** 2021-11-26

**Authors:** Qing-Hai Wang, Xing Gao, Hong-Song Yu, Ze Zhang, Quan-You Yu

**Affiliations:** 1 School of Life Sciences, Chongqing University, Chongqing 400044, China; wqh1226823@163.com (Q.-H.W.); gxing202026021022@163.com (X.G.); zezhang@cqu.edu.cn (Z.Z.); 2 School of Basic Medical Sciences, Zunyi Medical University, Zunyi 563000, China; yuhongsong@163.com

**Keywords:** sex pheromone, aldehyde synthesis and metabolism, terminal pathway, silkworm

## Abstract

**Simple Summary:**

Insect sex pheromone biosynthesis has received widespread attention, while the terminal pathway related to aldehyde synthesis and metabolism is still poorly understood at a molecular level. Previous studies found that the silkworm, *Bombyx mori* (Lepidoptera, Bombycidae), has two pheromone compounds, bombykol and bombykal, with a ratio of 11:1, while its closest wild relative, *B. mandarina*, only uses bombykol as a pheromone. In this study, sex pheromone gland transcriptomes were compared between the domestic and wild silkworms. All the candidate gene families were identified. Then we used the differentially expressed information, tissue and developmental expression profiles, and phylogenetic analysis to identify the putative causal genes involved in the terminal pathway. Our findings provide insights into the aldehyde synthesis and metabolism pathways and evolutionary conservation in moths.

**Abstract:**

Sex pheromones are vital to sexual communication and reproduction in insects. Although some key enzymes in pheromone production have been well studied, information on genes involved in the terminal pathway is limited. The domestic silkworm employs a pheromone blend containing (*E*,*Z*)-10,12-hexadecadienol (bombykol) and analogous (*E*,*Z*)-10,12-hexadecadienal (bombykal); whereas, its wild ancestor *B. mandarina* uses only bombykol. The two closely related moths might be a good model for exploring the genes involved in aldehyde pheromone synthesis and metabolism. By deep sequencing and analyzing the sex pheromone gland (PG) transcriptomes; we identified 116 candidate genes that may be related to pheromone biosynthesis, metabolism, and chemoreception. Spatiotemporal expression profiles and differentially expressed analysis revealed that four alcohol oxidases (*BmorAO1*; *2*; *3*; and *4*); one aldehyde reductase (*BmorAR1*); and one aldehyde oxidase (*BmorAOX5*) might be involved in the terminal pathway. Phylogenetic analysis showed that, except for *BmorAO3* and *MsexAO3*, AOs did not show a conversed orthologous relationship among moths; whereas, ARs and AOXs were phylogenetically conserved. This study provides crucial candidates for further functional elucidation, and which may be utilized as potential targets to disrupt sexual communication in other moth pests.

## 1. Introduction

Sex pheromones are usually biosynthesized and released from female moths using a specialized pheromone gland (PG) located at the tip of the female’s abdomen [1]. Species-specific pheromones play a vital role in intraspecific sexual communication and interspecific reproductive isolation [2]. To date, a large number of sex pheromones have been chemically identified from more than 1600 moth species [3]. Most known pheromones (> 75%) belong to Type-I, i.e., even-numbered C_10_-C_18_ acyclic and unsaturated fatty acid-derived compounds, such as alcohols, aldehydes, and acetate esters [4]. To achieve reproductive isolation, even closely related moth species will have different components, or have the same components but in different blend ratios [5,6]. Thus, species-specific pheromones are often strictly controlled by the biosynthesis and metabolism pathway [6,7].

Due to the similar molecular scaffolds of sex pheromones, biosynthetic and metabolic pathways are relatively conserved in moths [6,7]. Generally, the biosynthetic pathway of the Type-I sex pheromone is regulated by the pheromone biosynthesis-activated neuropeptide (PBAN), released from the brain-subesophageal ganglion. PBAN can migrate to PGs, where it interacts with the PBAN receptor (PBANr) in the PG cell membrane [8]. The resulting signal then triggers a series of enzymatic reactions, starting with the de novo biosynthesis of pheromone precursors, mainly C_14_, C_16_, or C_18_ saturated fatty acids, using acetyl-CoA carboxylase (ACC) and fatty acid synthase (FAS) [9]. Then, one or more double bonds are introduced into the pheromone precursors by desaturase (DES) at some specific carbon locations [10,11,12,13]. The unsaturated pheromone precursors are transformed to corresponding fatty alcohols by fatty-acyl reductase (FAR). On the one hand, the unsaturated fatty alcohols can be used as pheromone components in some moths [14,15,16]. They might also be converted to aldehydes or acetate esters by alcohol oxidase (AO) [17] or acetyltransferase (ACT) [18]. However, the produced aldehydes might be partially metabolized to corresponding alcohols and carboxylic acids by aldehyde reductase (AR) [19] and aldehyde oxidase (AOX) [20]. Similarly, acetate esters might be degraded into alcohols by carboxylesterase (COE) [21].

The general pathway of sex pheromone biosynthesis has been continuously improved since the first identification of bombykol in *B. mori* [22,23,24]. In the past two decades, some enzyme and non-enzyme genes involved in bombykol production have been cloned and functionally characterized in *B. mori*, including *pgdesat1* [25,26], *pgFAR* [26,27], *BmFATP* [28], *pgACBP* [29], *mgACBP* [29], and several lipase genes [30]. Some of the homologous genes have also been identified in other moth species, such as *A. pernyi* [10], *O. nubilalis* [31,32], *Yponomeuta spp.* [33], *S. exigua* [34], and *E. japonica* [35]. Comparative analyses showed that the above-mentioned enzyme genes are often clustered into orthologous clades of sex pheromone synthesis in the phylogenetic trees [25,33,34,35], suggesting that they are highly conserved in moths.

The biosynthetic pathway of sex pheromones has received widespread attention, while the terminal pathway related to aldehyde synthesis and metabolism are still poorly understood at a molecular level. Enzyme activities of alcohol oxidase were found in the female PGs of *Manduca sexta* [36], *Hyphantria cunea* [37], *Heliothis virescens*, and *Heliothis zea* [38], which suggested that alcohol oxidase is responsible for metabolizing fatty alcohols into aldehydes. In recent years, the AO family has been identified incidentally in some moth PG transcriptomes [39,40,41]. Aldehyde reductase and aldehyde oxidase can metabolize fatty aldehydes and might also be involved in the terminal pathway. This speculation stems from the fact that ARs and AOXs were both distributed in PGs and antennae, and the antennal enzymes have degrading activity for aldehyde pheromones [42,43,44]. Earlier biochemistry studies found that antennal aldehyde oxidase from *M. sexta* [45], *Antheraea polyphemus* [20], and *B. mori* [20] can degrade bombykal. Recently, an AOX gene, *AtraAOX2*, has been cloned from the antennae of *Amyelois transitella*, which can degrade the main pheromone component, *Z*11,*Z*13-16:Ald [43].

The silkworm, *Bombyx mori* (Lepidoptera, Bombycidae), is an important beneficial insect, domesticated from the Chinese wild silkworm *B. mandarina* about 5000 years ago [46]. A previous study found that bombykol is the only pheromone component in *B. mandarina* [47], whereas the sex pheromone of the domestic silkworm has been determined to be a mixture of two compounds, bombykol and bombykal, with a ratio of 11:1 [47,48]. In this study, comparative transcriptomes of pheromone glands were conducted between the domestic and wild silkworms. The putative genes involved in pheromone biosynthesis and metabolism have been identified. Combining the differential expression analysis, spatiotemporal transcription profiles, and comparative genomics, the crucial candidate genes were revealed. Actually, bombykol and bombykal are used as sex pheromones by 11 and 25 species of moths, respectively (http://www.pherobase.com/ (accessed on 10 June 2021)). Our results would provide an important basis for understanding the role of terminal enzymes in regulating pheromone components and their ratios in moth pests.

## 2. Materials and Methods

### 2.1. Insect Rearing

The domestic silkworm (p50T strain) and wild silkworm (Chongqing, China) were used for the experiment in this study. Larvae of the domestic silkworms were reared on mulberry leaves in the laboratory conditions, 25 ± 1 °C, 70% ± 2% relative humidity (RH), and a photoperiod of 14 h light and 10 h dark. The larvae of the wild silkworms were reared on mulberry trees in the Plant Garden of Chongqing University under natural conditions until pupation. The pupae of *B. mori* and *B. mandarina* were separated by male and female, and placed in different boxes till eclosion.

### 2.2. Sample Collection

The pheromone glands were quickly excised from the 1-day-old virgin females after 3 h of photosensitization. PGs from 20 individuals were used for one sample. PG_D and PG_W represent the PGs of the domestic and wild silkworms, respectively. All the samples were kept in RNAlater (Qiagen, Hilden, Germany) and stored at –80 °C till RNA extraction.

For the expression pattern analysis, various tissues were collected in the domestic silkworms, including the antennae, heads, thoraxes, abdomens (without pheromone glands), wings, and legs of the adult females. In addition, PGs from different developmental stages were also collected, including the pupal PGs (from the 4th day to the 9th day), the virgin adult PGs (3-h, 24-h, and 48-h females after eclosion), and the 3-h, 6-h, and 9-h mated adult PGs.

### 2.3. cDNA Library Preparation and Illumina Sequencing

Total RNAs from PG_D and PG_W were extracted using the TransZol Up Plus RNA Kit (TransGene, Beijing, China), following the manufacturer’s instructions. A NanoDrop ND-2000 spectrophotometer (Thermo Fisher Scientific, Waltham, MA, USA) was used to check RNA purity and concentration according to the absorbance at 260 nm, and 1.0% agarose gel was used to monitor RNA degradation. The two sequencing libraries (PG_D and PG_W) were prepared by using the Illumina Gene Expression Sample Prep Kit. Briefly, mRNA from the two samples was purified using Oligo (dT) magnetic beads from 30 μg of pooled total RNA. Subsequently, the mRNA was sheared into short fragments in the presence of divalent cations in fragmentation solution at 94 °C for 5 min. The short fragments were used as templates for the first-strand cDNA synthesis. Then, the second-strand cDNA was generated using dNTPs, RNaseH, and DNA polymerase I. Short sequences were amplified by PCR and purified with the QIAquick PCR Purification Kit (Qiagen, Shanghai, China). Ultimately, the cDNA library preparations were sequenced on a HiSeq™ 2000 platform (Novogene, Tianjin, China).

### 2.4. Sequence Assembly and Annotation

To obtain the clean reads, the raw reads were filtered using NGS QC Toolkit v2.3.3 software (http://www.nipgr.res.in/ngsqctoolkit.html (accessed on 3 June 2020)), and adaptor sequences and low-quality sequences were removed, including > 10% of poly-N or > 50% of bases whose Phred quality scores were ≤ 5. Reference genome and gene model annotation files were downloaded from SilkDB v3.0 (https://silkdb.bioinfotoolkits.net/ (accessed on 10 September 2020)). The paired-end clean reads from PG_D and PG_W were aligned to the *B. mori* reference genome using HISAT2 [49], and then the StringTie program was used for assembly [50]. The resulting transcripts were performed by BLASTX against the non-redundant (nr) protein database in NCBI with an e-value less than 1.0E-5. Subsequently, GO (Gene Ontology) annotations were performed by the Blast2GO program (https://www.blast2go.com/ (accessed on 13 October 2020)). Meanwhile, using InterProScan v5.50 software (http://www.ebi.ac.uk/interpro/ (accessed on 13 October 2020)), the amino acid sequences from protein-coding transcripts performed homology alignments against the InterPro database (http://www.ebi.ac.uk/interpro/ (accessed on 15 October 2020)) to acquire more accurate annotated results. Ultimately, GO functional distributions were obtained using WEGO software (http://wego.genomics.org.cn/ (accessed on 16 October 2020)).

### 2.5. Sequence Acquisition and Phylogenetic Analysis

To understand the evolutionary conservation of the enzyme genes in the terminal pathway, some of the species containing at least one aldehyde as a pheromone were collected (Table S1). Homologous protein sequences of AOs, ARs, and AOXs were mainly retrieved from the literature (Table S2). It is worth noting that *M. sexta* shares bombykal with the silkworms. Due to no PG transcriptome data being released, homologous searches were performed by BLAST against the gene sequences based on the whole genome annotation in *M. sexta* downloaded from the RefSeq database (https://www.ncbi.nlm.nih.gov/refseq/ (accessed on 20 May 2021)). In the silkworm, the candidate terminal enzymes were identified by a BLAST search against the transcripts of the PG transcriptomes. All the candidate enzyme genes are checked in the PFAM database (http://pfam.xfam.org/ (accessed on 25 May 2021)) to see whether they contain the corresponding domains. All the candidate genes assembled in the transcriptome have a one-to-one correspondence with SilkDB v3.0 (https://silkdb.bioinfotoolkits.net/ (accessed on 25 May 2021)) and SilkBase (http://silkbase.ab.a.u-tokyo.ac.jp/ (accessed on 25 May 2021)).

The alignments of multiple sequences were performed by MAFFT program (https://mafft.cbrc.jp/alignment/software/ (accessed on 2 June 2021)). The optimum phylogenetic models were calculated by ModelTest-NG v0.1.6 [51]. Maximum likelihood (ML) trees were reconstructed for AOs, ARs and AOXs using the RAxML-NG v1.0.0 program [52]. Branch supports were surveyed by bootstrapping 500 replicates, and bootstrap values (≥ 70%) were shown at the nodes.

### 2.6. Reverse Transcription-Polymerase Chain Reaction (RT-PCR) Analysis

Single-stranded cDNA was synthesized using 2 μg of total RNA from each sample using a PrimeScript^TM^ reagent Kit with gDNA Eraser (TaKaRa, Dalian, Liaoning, China), following the instruction in the manual. Gene-specific primers were designed using Primer Premier 5.0 software (Table S3). RT-PCR programs were 98 °C for 2 min, followed by 25–32 cycles of 98 °C for 15 s, 52–58 °C for 10 s, and 72 °C for 1 min. A total volume of 10 μL PCR reaction system was performed containing 8.4 μL of Golden Star T6 Super PCR Mix (TsingKe, Beijing, China), 0.3 μL of forward primer (10 mM), 0.3 μL of reverse primer (10 mM), and 1.0 μL of cDNA templates (200 ng/μL). Amplification products were analyzed on 1.0% agarose gels. *RpL3* (*ribosomal protein L3*) was used as an endogenous control gene to determine the consistency of the cDNA template concentration.

### 2.7. Quantitative Real-Time PCR (qRT-PCR) Analysis

Specific primers were designed and were listed in Table S4. *RpL3* was used as an internal reference gene for sample normalization. The mRNA expression levels were checked using the QuantiNova SYBR Green PCR Kit (Qiagen, Shanghai, China). The PCR amplified conditions were 95 °C for 2 min, followed by 40 cycles of 95 °C for 5 s, and 58 °C for 10 s. Each sample was measured in three independent biological replicates, and relative mRNA expression levels were calculated using the 2^−ΔΔCt^ method [53].

## 3. Results

### 3.1. Transcriptome Sequencing, Assembly, and Functional Annotation

RNA-sequencing (RNA-Seq) was performed on the sex pheromone glands of *B. mori* (PG_D) and *B. mandarina* (PG_W). Approximately 47.4 and 44.9 million raw reads were generated (Table S5). After removing the adaptors and low-quality sequences, the clean reads were assembled, resulting in 19,828 unique transcripts, of which 13,800 unigenes showed expression signals (RPKM ≥ 1) in at least one of the two samples. The expressed unigenes were searched for homology in the nr and InterPro database. Based on the BLAST and InterProScan results, Gene Ontology annotations were conducted by the Blast2GO program. Three functional categories were assigned for the expressed unigenes (Figure S1). In the cellular component category, the expressed unigenes were mostly enriched in the cell (GO: 0005623) and cell part (GO: 0044464). In the molecular function category, the most enriched GO terms were the catalytic activity (GO: 0003824) and binding (GO: 0005488). In the biological process category, the most enriched were the metabolic process (GO: 0008152) and the cellular process (GO: 0009987) (Figure S1).

### 3.2. Differentially Expressed Genes (DEGs) between Bombyx mori and Bombyx mandarina

**Figure 1:**
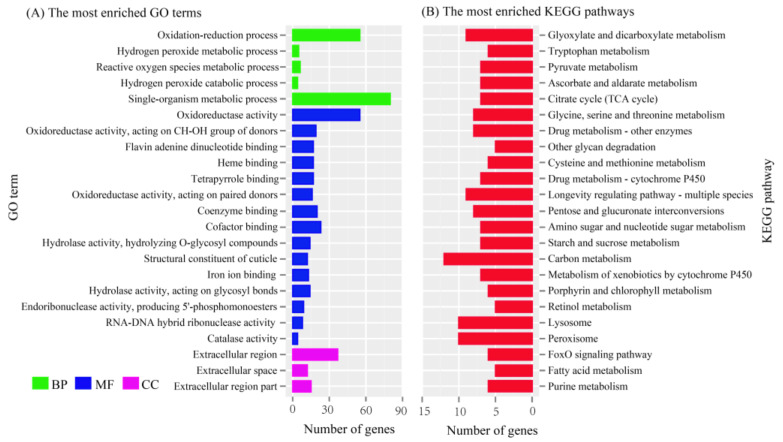
GO (**A**) and KEGG (**B**) enrichments of the differentially expressed genes (DEGs). GO: Gene Ontology; KEGG: Kyoto Encyclopedia of Genes and Genomes; BP: biological process; MF: molecular function; CC: cellular component.

**Figure 2:**
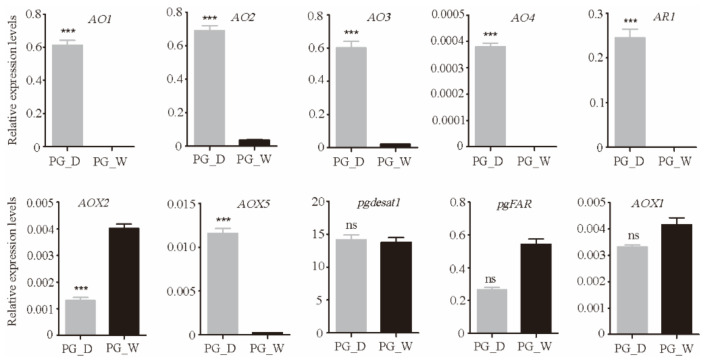
The qRT-PCR analysis of sixteen crucial candidate genes in the PGs (including seven DEGs and three non-DEGs). PG_D: *B. mori* PGs; PG_W: *B. mandarina* PGs; ns: no significance; *** *p* < 0.001, Student’s t-test.

The significant DEGs between the domestic and wild silkworms were identified with a strict false discovery rate, (FDR) < 0.001, and an absolute value of log2 fold-change (PG_D/PG_W) ≥1. In total, 1382 DEGs were identified in the PGs, including 838 up- and 544 down-regulated genes in the domestic silkworm (Figure S2). Blast2GO software was used for GO enrichment analysis of the differentially expressed genes. They were mainly enriched in the oxidation–reduction process, the single-organism metabolic process, in oxidoreductase activity, and in the extracellular region (Figure 1A). KEGG enrichment was conducted via KOBAS 3.0 (http://kobas.cbi.pku.edu.cn/anno_iden.php (accessed on 16 October 2020)). The most enriched pathways were glyoxylate and dicarboxylate metabolism, carbon metabolism, lysosome, and peroxisome (Figure 1B). The functional annotations of the DEGs indicated that there were many differences between the PGs of the domestic and wild silkworms in metabolic-related processes. To verify the reliability of transcriptome data, we checked the expression levels of sixteen candidate genes by qRT-PCR, including seven DEGs and three non-differentially expressed genes (Figure 2). The results indicated that the expression levels detected by qRT-PCR and RNA-Sequencing were comparable (Figure 2, Table S6).

### 3.3. *Identification of Candidate Genes involved in Pheromone Biosynthesis and Metabolism*

Based on the homologous BLAST searches, we identified 93 putative candidate genes that encode for the following proteins: acetyl-CoA carboxylase (ACC, *n* = 1), fatty acid synthase (FAS, *n* = 2), desaturase (DES, *n* = 8), fatty acid transport protein (FATP, *n* = 6), acyl-CoA-binding protein (ACBP, *n* = 2), lipase (LIP, *n* = 16), fatty-acyl reductase (FAR, *n* = 18), alcohol oxidase (AO, *n* = 25), aldehyde reductase family (AR, *n* = 11), and aldehyde oxidase (AOX, *n* = 4) (Table 1, Table S6). The results indicated that most families have multiple gene members expressed in the PGs.

**Figure 3:**
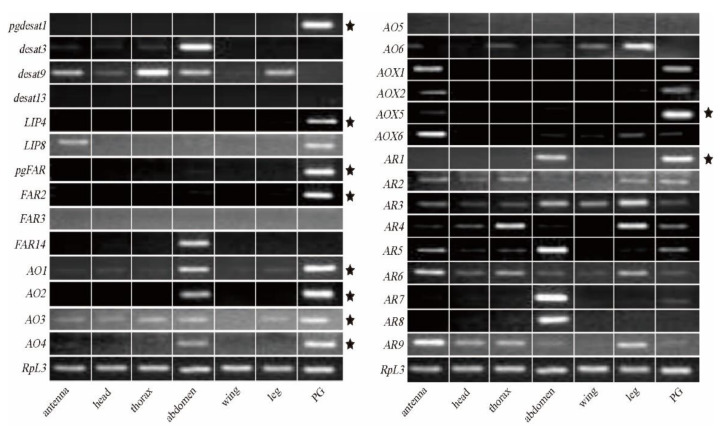
Expression patterns of sex pheromone biosynthesis-related genes in adult tissues. The RT-PCR products were detected by agarose gel electrophoresis. *RpL3* was used as an internal reference gene. Genes with an asterisk indicate a specific or biased expression in the PGs.

**Figure 4:**
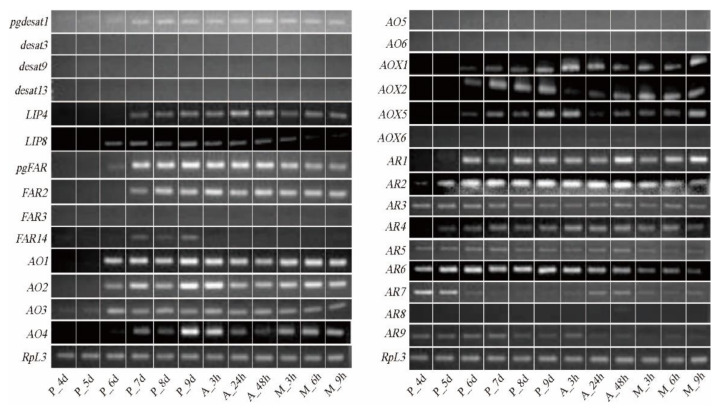
Expression patterns of sex pheromone biosynthesis- and metabolism-related genes in the PGs at different developmental stages. The RT-PCR products were detected by agarose gel electrophoresis. PG scheme P_4d to P_9d: the PGs from the 4th to 9th day of the pupae; A_3h, A_24h and A_48h: the virgin PGs from the 3-h, 24-h and 48-h old adults; M_3h, M_6h and M_9h: the 3-h, 6-h, and 9-h mated adult PGs.

**Figure 5:**
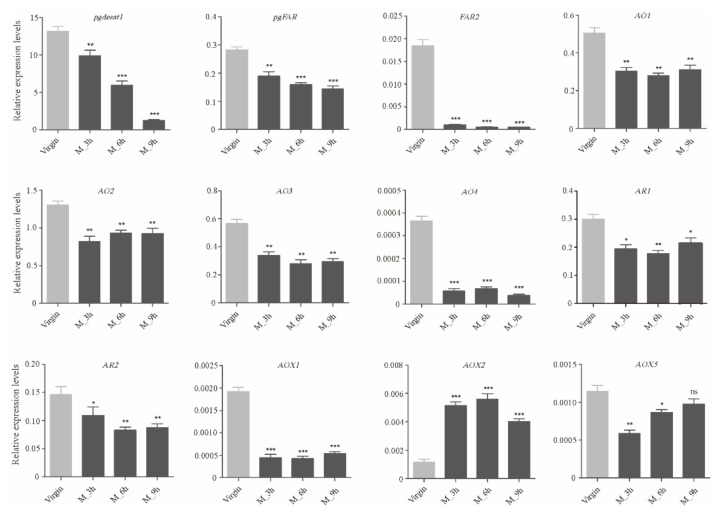
Effects of mating on expression of important candidate genes. The asterisk indicates significant difference between the virgin and mated PGs (ns: no significance; *p* < 0.05, “*”; *p* < 0.01, “**”; *p* < 0.001, “***”, Student’s t-test).

Since bombykol was first identified in *B. mori* in 1959 [14], the general pathway of sex pheromone biosynthesis has been established [22,23,24]. Several key genes have been cloned and functionally characterized in the silkworm, including *pgdesat1* [25,26], *pgFAR* [26,27], *BmFATP* [28], *pgACBP* [29], *mgACBP* [29], and four lipase genes [30]. These reported key genes showed abundant expression levels in the PGs (Table 1, Table S6). In particular, *pgdesat1* and *pgFAR* are the two most studied genes [25,26,27]. Compared with other desaturases (RPKM < 30) and FARs (most RPKM < 20), their expression levels reached 7984.24 and 215.88, respectively (Table 1, Table S6). Tissue expression profiles indicated that *pgdesat1* and *pgFAR* were specifically expressed in PGs (Figure 3) and began to be expressed in day 7 pupae (Figure 4). Several studies showed that sexual mating can not only down-regulate gene expression but also result in the termination of sex pheromone production [54,55,56]. We found that *pgdesat1* and *pgFAR* displayed significantly down-regulated expression after mating (Figure 5). In general, the causal genes involved in the pheromone biosynthesis often have characteristic expression profiles, which may provide an important reference for the identification of the genes involved in the terminal steps of the biosynthetic and metabolic pathways.

### 3.4. Genes involved in the Terminal Pathway of Bombykal Synthesis and Metabolism

It was suggested that AOs, ARs, and AOXs might play roles in aldehyde pheromone synthesis and metabolism [19,20,38]. In this study, we comprehensively characterized the putative causal genes in the terminal pathway and revealed the phylogenetic relationships with other moths.

#### 3.4.1. Alcohol Oxidase (AO)

Early studies have shown that alcohol oxidase is responsible for the conversion of fatty alcohol into analogous aldehyde pheromones [57]. In the present study, we identified 25 AOs in the PG transcriptomes, of which six genes showed differential expression between the domestic and wild silkworms (Table S6). For the six DEGs, five of them were up-regulated in the domestic silkworm, and *BmorAO1*, *2*, and *3* were highly expressed (Table S6). Expression profiles of all the six differentially expressed AO genes were detected in the various tissues of the female adults (Figure 3). *BmorAO1*, *2*, and *4* were specifically expressed in PGs and abdomens (without pheromone glands). *BmorAO3* showed high expression in PGs and was expressed at low levels in the other five tissues. However, the differentially expressed *BmorAO5* and *6* were not detected expression signals in the domestic silkworm PGs (Figure 3). We also found that *BmorAO1*, *2*, *3*, and *4* began to be expressed in the PGs in day 6 or 7 pupae (Figure 4), and were down-regulated after sexual mating (Figure 5). Taken together, *BmorAO1*, *2*, *3*, and *4* might be the putative causal genes in the process of *B. mori* catalyzing bombykol to bombykal.

To understand the evolutionary relationship of AO genes among moths, a maximum likelihood (ML) phylogenetic tree was constructed (Figure S3). Most of the AO protein sequences were retrieved from the published PG transcriptome data [39,40,41,58]. *Manduca sexta* shares bombykal with the silkworm, and its whole genome was released. The genome-wide identification of the AO gene family was also conducted in *M. sexta*. The ML tree indicated that most of the putative causal genes (*BmorAO1*, *2*, *3*, and *4*) had no clear orthologous genes. Only *BmorAO3* and *MsexAO3* were orthologous genes, and their sequence identity was 72.6%. This result indicates that pheromone biosynthesis AOs may not be as conserved in moths as the key genes have been reported.

#### 3.4.2. Aldehyde Reductase (AR)

**Figure 6:**
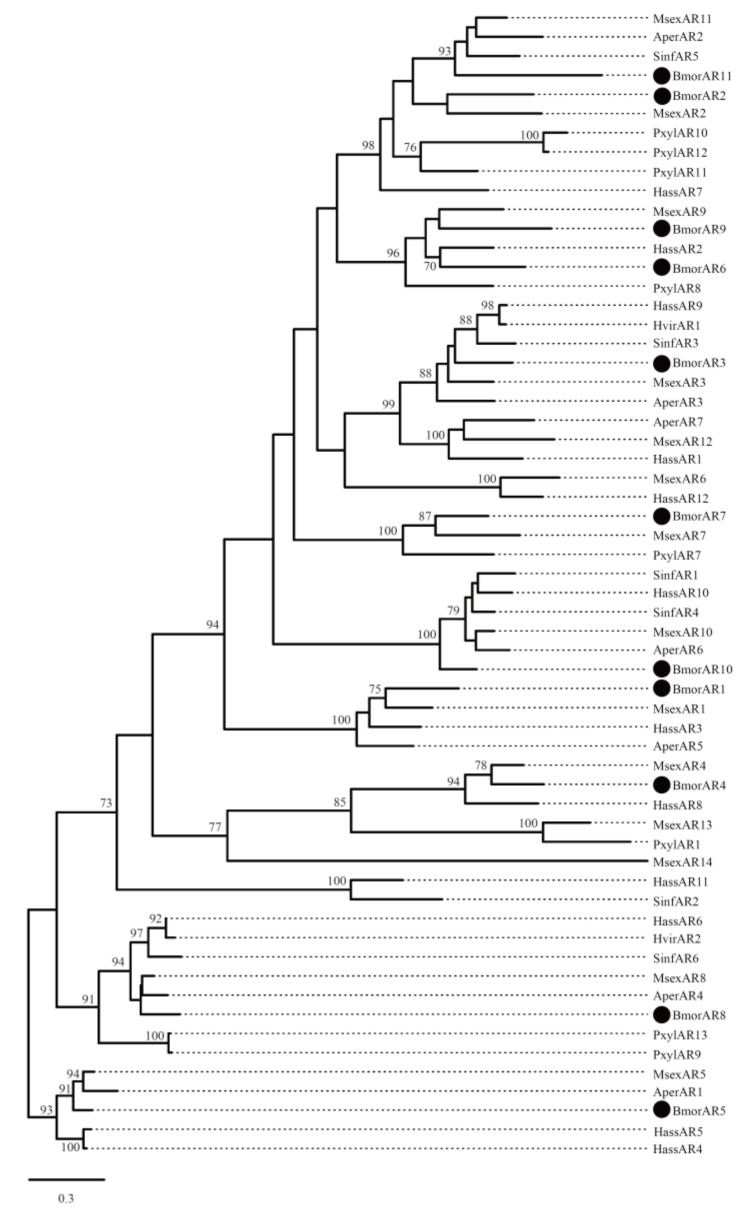
Phylogenetic tree of insect aldehyde reductases (ARs). Other moth AR proteins were given in Table S2. The *B. mori* genes are marked with a black circle. Bootstrap values ≥ 70% are shown.

In the terminal pathway, aldehyde reductase is mainly responsible for changing aldehyde to alcohol, which is a reverse process of alcohol oxidase [42]. Eleven ARs were identified in the PGs (Table S6). *BmorAR1* showed differential expression between the domestic and wild silkworm and was highly expressed (RPKM = 582.61) in the PGs of *B. mori* (Table S6). Meanwhile, *BmorAR1* displayed a PG-biased expression (Figure 3) and began to express in the PGs in day 6 of pupae (Figure 4). Furthermore, its transcriptional level was significantly decreased in the mated PGs compared with the virgin PGs (Figure 5). All the experimental datasets suggested that *BmorAR1* might be the key gene responsible for the reduction of bombykal in the pheromone gland. Interestingly, the phylogenetic analysis found that *BmorAR1, MsexAR1*, *HassAR3*, and *AperAR5* clustered in a separated clade (Figure 6). Their protein sequence identities reached 53−57%. Thus, aldehyde reductase involved in the terminal pathway might be relatively conserved in moths.

#### 3.4.3. Aldehyde Oxidase (AOX)

Aldehyde oxidases (AOXs) are molybdo-flavoenzymes that oxidize aliphatic/aromatic aldehydes into the corresponding carboxylic acids [44,59]. In insects, many studies have focused on the odorant degradation of AOXs in the antennal tissue, including pheromone and aldehyde signaling molecules from host plants [43,44,45]. It is speculated that AOXs in the pheromone gland may participate in the degradation of aldehyde pheromone and regulate its content [60]. In the silkworm, eight AOX genes have been identified in the whole genome [61,62]. In our PG transcriptome datasets, four AOXs showed expression signals, namely *BmorAOX1*, *2*, *5*, and *6* (Table S6). *BmorAOX2* and *BmorAOX5* showed differential expression, and both of them were up-regulated in the domestic silkworm (Figure 2, Table S6). Tissue expression pattern analysis indicated that *BmorAOX5* was specifically expressed in *B. mori* PGs, and *BmorAOX1* and *BmorAOX2* were specifically expressed in the antennae and PGs (Figure 3). Meanwhile, *BmorAOX1*, *2*, and *5* began to express in PGs at day 6 of pupae development (Figure 4), and *BmorAOX1* and *BmorAOX5* were down-regulated after mating (Figure 5). A previous study found that *BmorAOX5* can metabolize multiple aromatic aldehydes and fatty aldehydes in vitro [60]. Combining the spatiotemporal expression patterns and down-regulation after mating, we suggested that the differentially expressed *BmorAOX5* might be the putative causal gene mediating content of bombykal in the PGs of the domestic silkworm.

**Figure 7:**
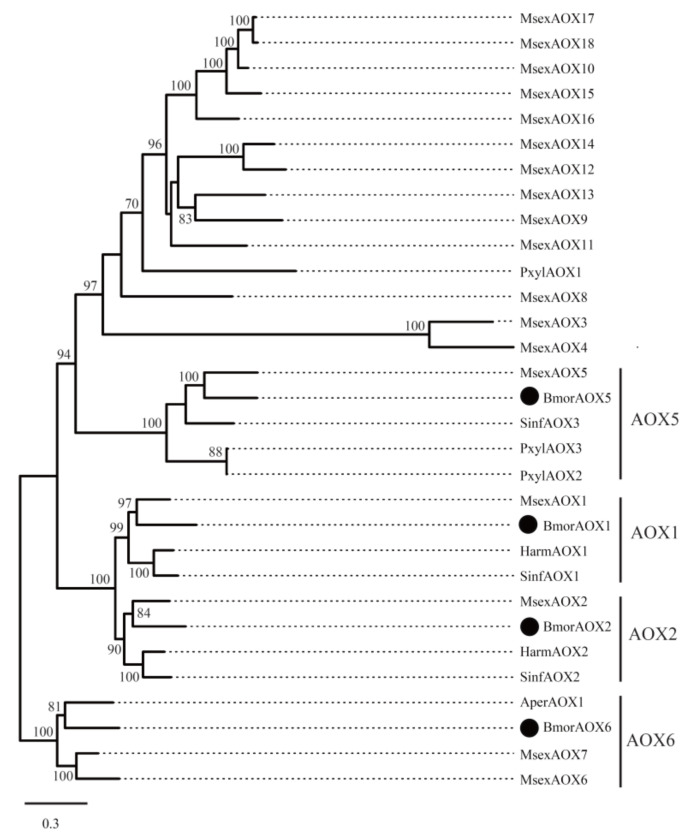
Phylogenetic tree of insect aldehyde oxidases (AOXs). Other moth AOX proteins were given in Table S2. The *B. mori* genes are marked with a black circle. Bootstrap values ≥ 70% are shown.

Similarly, the phylogenetic tree of AOXs was also constructed (Figure 7). It was indicated that the four silkworm AOXs and other moth AOXs were clustered into four different clades, namely, AOX1, AOX2, AOX5, and AOX6. For instance, in the AOX5 clade, *BmorAOX5*, *MsexAOX5*, and *SinfAOX3* showed a 1:1:1 orthologous relationship. It was suggested that the AOX family is relatively conserved during evolution. This may provide an important basis for revealing the universal mechanism of moth insects regulating the content of aldehyde pheromones.

## 4. Discussion

In moth species, the general pathway of sex pheromone biosynthesis has been established since the first identification of bombykol in 1959 [14]. In this study, earlier verified genes were characterized again in the silkworm, such as *pgdesat1*, *pgFAR*, *BmFATP*, etc. They often presented specific and high expression in the PGs (Figure 3, Table S6). Those key genes showed no differential expression between the domestic and wild silkworms (Table S6). This may be because they are involved in the synthesis of pheromone precursors and bombykol, and in these components, there is no difference between *B.mori* and *B. mandarina*. In addition, two FAR genes exhibited different preferences for C_14_ and C_16_ fatty acid substrates in *Spodoptera exigua* [34]. In the silkworm, *pgFAR* is responsible for catalyzing the biosynthesis of bombykol [27]. Based on our experiments, another PG-specific *FAR2* has great consistency with *pgFAR*, such as abundant expression in adult PGs (RPKM = 255.69), initiation of expression in late pupal PGs, and down-regulation after mating (Figure 3, Figure 4, Figure 5, Table S6). Except for *pgFAR*, whether the silkworm also has a second functional FAR involved in the synthesis of bombykol deserves further study.

*B. mori* females produce two sex pheromone components in their PGs, the major bombykol and the minor bombykal pheromones with a ratio of 11:1, whereas *B. mandarina* females utilize only bombykol as a pheromone [47,48]. In this study, we found that those genes related to bombykol synthesis and the early steps in the pathway have no expression differences between the domestic and wild silkworms (Table S6). We suspected that the difference in the pheromone component may be caused by the terminal pathway. Previous studies indicated that alcohol oxidase is involved in the conversion of fatty alcohol into analogous aldehyde pheromone [37,57]. It has also been found that alcohol oxidase can catalyze the production of (9*Z*,12*Z*,15*Z*)-9,12,15-octadecatrienal from ^13^C-labeled linolenyl alcohol, and can also catalyze other C_18_ and C_19_ fatty alcohols to produce corresponding aldehydes in the pheromone gland of *Hyphantria cunea* [37]. In this study, four differentially expressed alcohol oxidase genes (*BmorAO1*, *2*, *3*, and *4*) were suggested as bombykol-to-bombykal conversion enzymes (Figure 3, Figure 4, Figure 5), which were involved in the terminal pathway. Since fatty-acyl desaturase and fatty-acyl reductase are highly conserved in the moth species [25,33,34], we are interested in the evolutionary conservation of enzyme genes involved in the terminal steps. Based on the phylogenetic analysis of AO genes in moths containing aldehyde pheromone (Figure S3), it was shown that the putative causal AO genes had no orthologous genes with other species, except for *BmorAO3* and *MsexAO3*. Is it because both *B. mori* and *M. sexta* contain bombykal? Whether the conserved *AO3* orthologous gene is involved in the bombykal synthesis of the two species is worthy of further functional verification.

Previous studies suggested that aldehyde pheromones might be metabolized to corresponding alcohols and carboxylic acids by aldehyde reductase and aldehyde oxidase, respectively [42,44,59]. In this study, *BmorAR1* and *BmorAOX5* were identified as the putative bombykal-metabolizing enzyme genes in the silkworm PGs (Figure 3, Figure 4, Figure 5). It is worth noting that one AR gene (*AKR2E5*), corresponding to *BmorAR4* in this study, was cloned from the fat body on day 3 fifth-instar larvae, which can reduce carbonyl compounds in vitro, such as bombykal and 11-hexadecenal [42]. However, it was expressed only at low levels in the PGs of *B. mori* (RPKM = 4.52) and *B. mandarina* (RPKM = 10.41) and showed abundant expression levels in the thoraxes and legs (Figure 3), suggesting that *BmorAR4* (*AKR2E5*) may function in other tissues instead of bombykal reductase in the PGs. In addition, aldehyde pheromones are biosynthesized and released in many moths, such as *B. mori*, *A. pernyi*, *H. virescens*, *H. armigera*, *H. assulta*, *P. xylostell* and *S. inferens* (Table S1). Phylogenetic trees were reconstructed for the AR and AOX genes in these species (Figure 6 and Figure 7). *BmorAR1* and its orthologous AR genes were grouped into a clade (Figure 6), and *BmorAOX5*, *MsexAOX5*, *SinfAOX3*, *PxylAOX3*, and *PxylAOX2* formed a monophyletic clade (Figure 7). Thus, aldehyde-metabolic AR and AOX genes may be relatively conserved in moths.

Taken together, four AOs, *BmorAR1*, and *BmorAOX5* were identified as the candidate enzyme genes involved in the synthesis and metabolism of bombykal in *B. mori*. Based on the present results, we are still not sure which one or more AOs play the role of converting bombykol to bombykal. At the same time, we do not know whether *BmorAR1* and *BmorAOX5* play a key role in the difference in bombykal composition between the domestic and wild silkworms. These questions need to be verified by genomic data, subsequent heterologous expression, enzyme activity analysis, and other experiments, keeping in mind the choice of cloning and heterologous protein expression. Generally, the process of sex pheromone synthesis and metabolism is actually a special aspect of fatty acid synthesis and metabolism. Fatty acid synthesis and metabolism are the basic life processes in organisms such as bacteria and insects. It is not excluded that some of the genes in sex pheromone synthesis pathway in insects are evolutionarily conserved with the homologs in bacteria, and therefore represent a possible result of common evolution between the moth sex pheromone gland and bacterial symbionts that would eventually be capable of pheromone biosynthesis.

## 5. Conclusions

Comparative PG transcriptomes were conducted in the domestic and wild silkworms. The candidate genes involved in pheromone biosynthesis and metabolism were identified. Based on the tissue and developmental expression profile analyses, *BmorAO1*, *BmorAO2*, *BmorAO3*, *BmorAO4*, *BmorAR1*, and *BmorAOX5* were characterized as the putative causal genes involved in the terminal pathway of bombykal synthesis and metabolism in the silkworm. Phylogenetic analysis indicated that AOs showed higher divergence among moths, and ARs and AOXs were relatively conserved during evolution. This study revealed valuable information about the terminal pathway of pheromone synthesis and metabolism, and provided important candidates for functional verification.

## Figures and Tables

**Table 1:** Important candidate genes related to sex pheromone biosynthesis and metabolism.

Gene Name (Displayed/Total)	RPKM_D	RPKM_W	DEG	Functional Annotation
*ACC1* (1/1)	21.40	21.94	*n*	acetyl-CoA carboxylase
*FAS1* (1/2)	284.73	175.52	*n*	fatty acid synthase
*pgdesat1* (1/8)	7984.24	7534.97	*n*	acyl CoA desaturase
*BmFATP* (1/6)	125.52	289.58	*n*	fatty acid transport protein
*ACBP1* (2/2)	31.77	19.69	*n*	acyl-CoA-binding domain-containing protein
*ACBP2*	43.40	53.56	*n*	acyl-CoA-binding domain-containing protein
*LIP1* (4/16)	234.39	131.44	*n*	lipase 3
*LIP2*	211.95	243.04	*n*	lipase 3-like
*LIP3*	81.45	94.26	*n*	lipase member H-A
*LIP4*	44.16	6.02	y	lipase member H
*pgFAR*(2/18)	215.88	734.50	*n*	fatty-acyl reductase
*FAR2*	255.69	248.61	*n*	putative fatty acyl-CoA reductase
*AO1* (3/25)	891.26	2.23	y	15-hydroxyprostaglandin dehydrogenase
*AO2*	925.77	93.66	y	15-hydroxyprostaglandin dehydrogenase
*AO3*	1036.26	124.93	y	carbonyl reductase [NADPH] 3
*AR1* (2/11)	582.61	66.52	y	aldo-keto reductase AKR2E4-like
*AR2*	337.63	718.81	*n*	aldo-keto reductase AKR2E4-like
*AOX1* (3/4)	357.31	318.79	*n*	aldehyde oxidase 1
*AOX2*	48.97	7.67	y	xanthine dehydrogenase-like
*AOX5*	33.06	5.21	y	xanthine dehydrogenase/oxidase

RPKM: Reads per kilobase per million mapped reads; RPKM_D: expression level in the domestic silkworm; RPKM_W: expression level in the wild silkworm. “y” and “*n*” mean differentially expressed gene (DEG) and non-differentially expressed gene. Functional annotation was obtained with a BLAST search against the nr database in NCBI.

## Data Availability

The protein sequences of the identified genes were included in Table S6. All published data are available upon formal request.

## Supplementary Materials

The following are available online at https://www.mdpi.com/article/10.3390/insects12121062/s1, Figure S1: Histogram of gene ontology (GO) classification; Figure S2: The volcano plot of the differentially expressed genes (DEGs); Figure S3: Phylogenetic tree of insect alcohol oxidases (AOs). Sequences of AOs are given in Table S2. The *B. mori* genes are marked with a black circle. Bootstrap values ≥7 0% are shown; Table S1: Sex pheromone components in the representative moths. *n*/a: not applicable; Table S2: Homologous proteins from other insects for phylogenetic analysis; Table S3: RT-PCR primers for the putative candidate genes; Table S4: qRT-PCR primers for the crucial candidate genes; Table S5: Summary of the clean reads mapped to the silkworm reference genome; Table S6: Identification of putative candidate genes related to pheromone biosynthesis and degradation. The accession numbers of the candidate enzyme genes in SilkDB v3.0 (https://silkdb.bioinfotoolkits.net/) and SilkBase (http://silkbase.ab.a.u-tokyo.ac.jp/) were listed.
