# Supplementary material for: Exploring the Terminal Pathway of Sex Pheromone Biosynthesis and Metabolism in the Silkworm
Source: Insects. 2021 Nov 26;12(12):1062. doi: 10.3390/insects12121062 (PMC8706005; doi:10.3390/insects12121062)
Supplement: Supplementary file 1 [file insects-12-01062-s001.zip › Supplementary Files/Table S5.pdf]

Table S5. Summary of the clean reads mapped to the silkworm reference genome

Sample name	PG_D	PG_W
Total clean reads	47441872	44929938
Read-1 mapped	14647408	14220087
Read-2 mapped	14500599	14133324
Total mapped	29148007	28353411
Mapped ratio	61.44%	63.11%
