# Supplementary figures and images for: Exploring the Terminal Pathway of Sex Pheromone Biosynthesis and Metabolism in the Silkworm
Source: Insects. 2021 Nov 26;12(12):1062. doi: 10.3390/insects12121062 (PMC8706005; doi:10.3390/insects12121062)

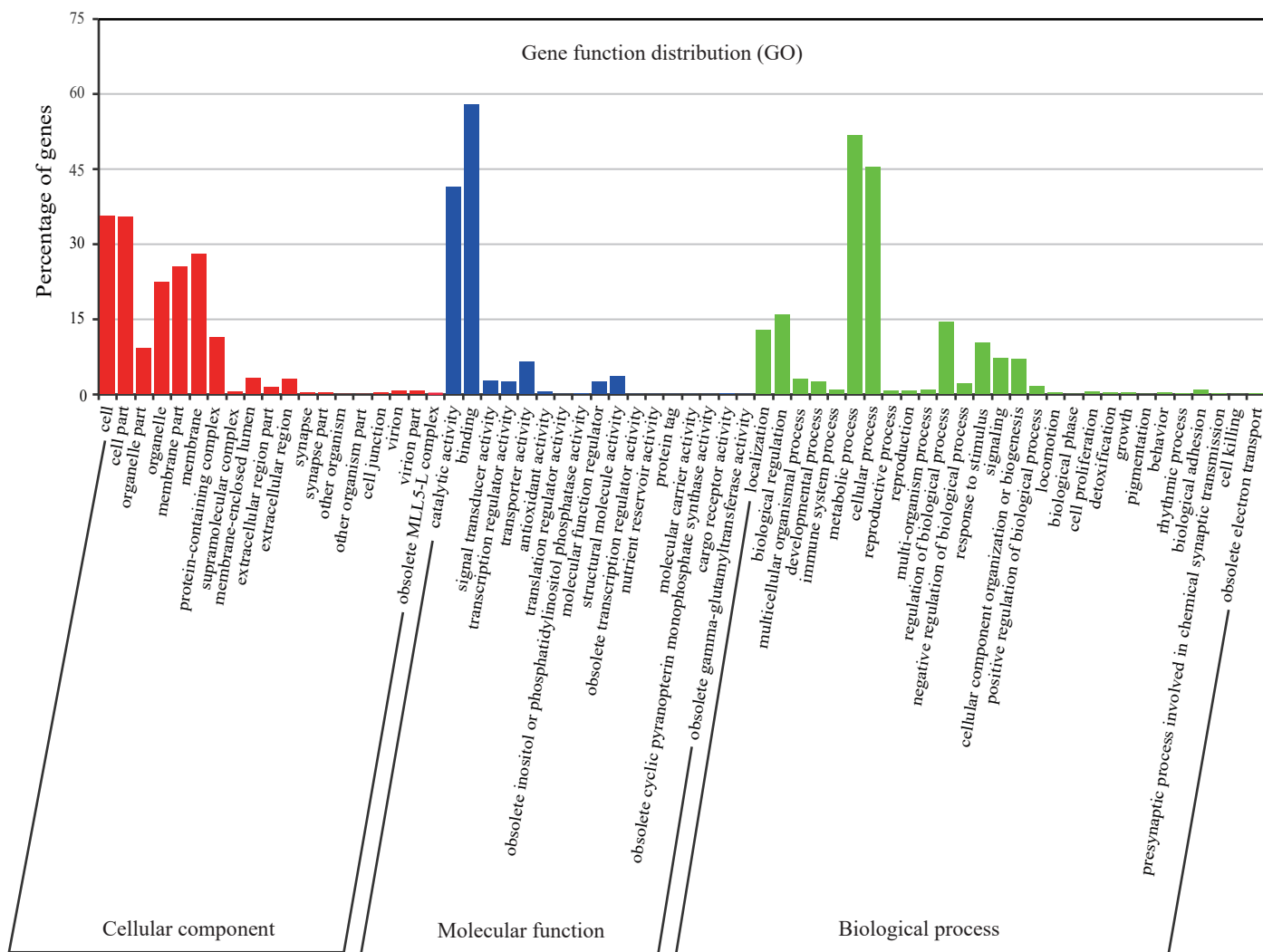

Supplement: Supplementary file 1 [file insects-12-01062-s001.zip › Supplementary Files/Figure S1.pdf]

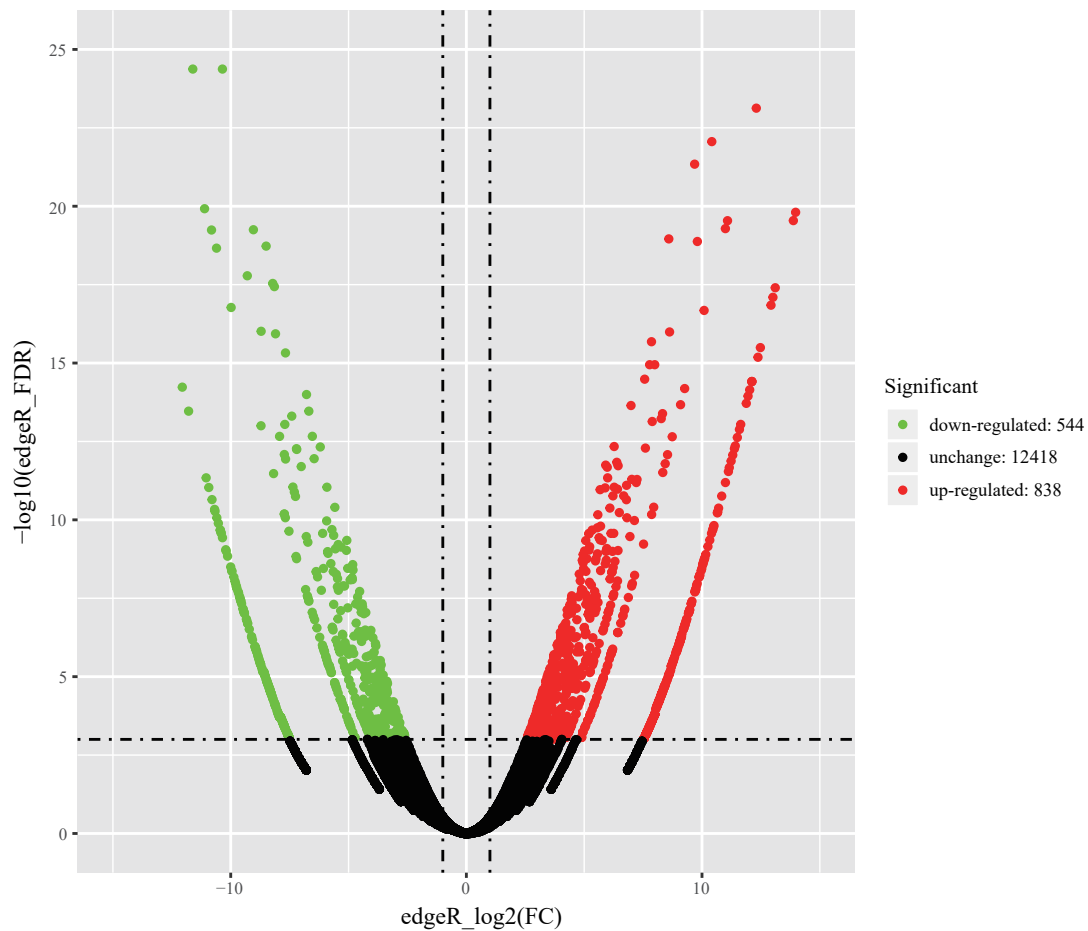

Supplement: Supplementary file 1 [file insects-12-01062-s001.zip › Supplementary Files/Figure S2.pdf]

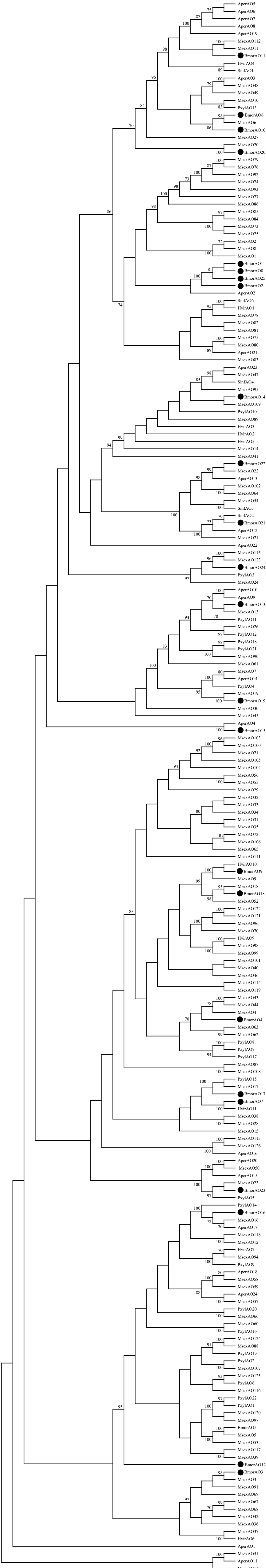

Supplement: Supplementary file 1 [file insects-12-01062-s001.zip › Supplementary Files/Figure S3.pdf]
